# Neurophysiology for Detection of High Risk for Psychosis

**DOI:** 10.1155/2016/2697971

**Published:** 2016-08-07

**Authors:** Lara N. Pantlin, Deana Davalos

**Affiliations:** Department of Psychology, Colorado State University, Campus Box 1876, Fort Collins, CO 80523, USA

## Abstract

Schizophrenia is a complex and often disabling disorder that is characterized by a wide range of social, emotional, and cognitive deficits. Increasing research suggests that the greatest social and cognitive therapeutic impact comes from early identification. The present study applied a well-established neurophysiological paradigm in the schizophrenia literature, mismatch negativity (MMN), to college students identified as high risk (HR) for psychosis to investigate MMN as a potential biomarker for the onset of psychosis. The hypothesis was that HR would exhibit attenuated MMN amplitudes compared to controls, as has been established in individuals with chronic schizophrenia. Participants (*N* = 121) were separated into Group 1 (controls) (*n*_1_ = 72) and Group 2 (HR) (*n*_2_ = 49) based on the established cutoff score of the 16-item Prodromal Questionnaire. Participants then completed a time based MMN paradigm during which brain activity was recorded with EEG. For all electrode locations, controls demonstrated significantly more negative amplitudes than HR (*Cz*: *F*(1,119) = 8.09, *p* = .005; *Fz*: *F*(1, 119) = 5.74, *p* = .018; *Pz*: *F*(1,119) = 5.88, *p* = .017). Results suggested that MMN may assist in identifying those who appear high-functioning but may be at risk for later development of psychosis or cognitive and psychological difficulties associated with psychosis.

## 1. Introduction

Recently, clinical research has focused on at-risk mental state (ARMS) populations or individuals in the prodromal stage of a mental illness. Prodromal is the term used to describe the early symptomatic stages of an illness or disease and is a critical developmental stage in terms of the development of psychosis. Psychosis is considered to be a core symptom of schizophrenia, with aspects of psychosis generally starting in adolescence and often progressing to the first psychotic break in young adulthood [1]. Recent research suggests, however, that there may be cognitive aspects of psychosis that could be identified much earlier in life which would likely lead to more effective interventions as cognitive and social dysfunctions emerge earlier and are thought to evolve to become the most disabling symptoms [1, 2].

Research in this area has aimed to develop tools that provide predictive information regarding early prevention methods [3, 4]. Neurophysiological measures, such as mismatch negativity (MMN), are considered to be ideal measures of brain functioning associated with psychopathology due to the fact that they are generally easy to record and often can assess the brain's responses to stimuli in the absence of attention or motivation [5]. For researchers studying clinical populations, the ability to measure how the brain processes information without having to worry about the confounding effects of attention or effort is ideal.

MMN has been described as an auditory event-related potential (ERP) that can be used to assess the brain's response to novel or deviant stimuli. It is considered to be an early response that is generated approximately 140–210 ms after a stimulus violates the regularity of recent auditory tones. This paradigm is presumed to be primarily generated in the auditory cortex and therefore is frequently applied to disorders associated with disturbances in sensory information processes, including individuals with schizophrenia [6–10].

Shelley et al. [11] were among the first to report attenuated MMN amplitudes in patients with schizophrenia. In a meta-analysis, Umbricht and Krljes [12] examined 40 studies and determined that those with chronic SZ consistently had reduced MMN amplitudes compared to healthy control (Cohen's *d* = .99). The reduced amplitudes in SZ are thought to be reflective of auditory disturbance, which is considered an underlying pathophysiological mechanism associated with the onset of psychosis and schizophrenia [3].

Umbricht and Krljes [12] also addressed different types of MMN (i.e., latency, duration, and frequency) and found that duration MMN (dMMN), which assesses the brain's ability to detect differences in timing stimuli, demonstrated an effect twice as large as MMNs that used frequency as the deviant. Other studies have also found duration MMNs to be unique in terms of the sensitivity to detect neurophysiological differences in individuals with SZ or their family members. Magno et al. [13] compared relatives of patients with first-episode or chronic patients with schizophrenia and found no effect for pitch but did find differences in dMMN amplitudes. Todd et al. [14] compared the different domains of MMN (duration, frequency, and intensity) in early and late stages of schizophrenia and also found strong differences for dMMN compared to other domains. Thus, dMMN has been referred to as one of the most robust and reliable evaluations of deficits in sensory information processing in schizophrenia [12, 13, 15].

The attenuated amplitude in MMN in patients with chronic schizophrenia (Cohen's* d* = .99, [12]) has given reason to believe that MMN may be a predictor of psychosis [15, 16].

Therefore, researchers in the field have used the MMN paradigm to compare first-episode, chronic, and high-risk family members of patients with SZ and healthy participants, and studies have resulted in conflicting findings. For example, when Salisbury et al. [10] compared healthy and first-episode patients, results suggested no differences in MMN amplitudes between these populations. However, follow-up longitudinal studies on these first-episode patients revealed significant reductions in MMN amplitudes in the 1.5 years following first hospitalization [10]. Other studies have also supported a significant difference between recent-onset or first-episode patients and healthy controls [7, 17, 18]. Jahshan et al. [19] also found reductions between recent-onset and healthy participants but more notably found significant differences between high-risk and healthy participants.

Following these reports, researchers began to compare participants considered ultra-high-risk (UHR) or prodromal to various other stages of SZ. Brockhaus-Dumke et al. [20] were among the first to demonstrate trends where patients with chronic SZ had the most reduced dMMN amplitudes, then prodromal subjects, and healthy controls. dMMN amplitudes have consistently demonstrated the most robust findings, especially when exploring the differences between a seemingly homogenous population. Murphy et al. [21] evaluated MMN deviants that differed in both duration and frequency in at-risk adolescents (ages 11–13). Findings revealed a significantly attenuated mean MMN amplitude for the at-risk group at frontal and temporal regions compared to controls. Atkinson et al. [22] also found a reduction in dMMN amplitudes when comparing UHR to patients who had experienced a first episode. At minimum, other reports have shown consistent trends [23], such that dMMN is thought to be reduced in these early stages of psychosis. Otherwise, there is seemingly limited research investigating MMN differences between clinically at-risk participants and healthy controls.

The limited research and conflicting nature of findings of these early reports may be due to difficulty associated with grouping this participant population; however, increasing the accuracy of identifying at-risk is the foundational step to establishing and improving prevention and early intervention techniques [24]. The line between controls and patients with schizophrenia is much more concrete than the line between controls and prodromal stages. Therefore, as the field has moved towards examining these early stages, survey measures have been established to evaluate the degree to which a person may be at risk or in the prodromal stages of psychosis [24, 25]. Schultze-Lutter and colleagues [25] address this issue through the use of multiple surveys to accurately identify those in the prodromal stages of psychosis. Their findings ultimately suggest that there are subgroups (early and late) within the prodromal stage and that using a combination of methods (i.e., multiple surveys) provides an additional degree of accuracy when classifying individuals. The continued emphasis and movement towards developing measures to identify and predict the onset of psychosis has led to applying neurophysiological tools, such as the MMN paradigm, paired with those at risk, in the hopes of creating better identification tools that lead to improved prevention and intervention methods.

Nagai et al. [16] claimed that features of the MMN paradigm provide support for use as a predictive tool to identify clinical stages of psychosis or furthermore provide risk assessment information regarding the likelihood that an individual may develop psychosis. These features include the following: The MMN paradigm is thought to be reflective of the N-methyl-d-aspartate receptor, preattentive auditory sensory memory, and the findings of attenuated MMN amplitudes are often found in patients with chronic SZ in literature. The present study aimed to further support these claims and argue for MMN as a potential early detection tool to identify biomarkers of schizophrenia and provide predictive information regarding the onset of psychosis. Duration MMN was used because of the robust findings in literature supporting dMMN as a tool to predict the likelihood of converting to psychosis [16]. The hypothesis was that participants who were categorized as high risk for developing psychosis based on the 16-item Prodromal Questionnaire would exhibit significantly attenuated MMN amplitudes at each electrode location compared to those who were not considered at risk.

## 2. Method

### 2.1. Participants

A survey screening for high risk for psychosis (16-item Prodromal Questionnaire,* DSM-IV *[26]) was administered to undergraduate students in an introductory level psychology course targeting mainly freshman and sophomore students at a large university. The screening was a part of a larger screening survey that also included demographic information (*N* = 121; mean age = 19.5; 57% female). Information regarding the participant or the participant's family history of psychological and/or neurological disorders was assessed via self-report in the survey. Participants were asked to indicate whether they or their first-degree family members had a history of psychological or neurological disorders. Participants who provided information that revealed psychiatric history of family members or self-history were excluded from the final analysis because this study did not aim to address issues of comorbidity.

Prior to data collection, a power analysis was run and revealed that in order to have 80% power each group would need at least 47 participants. Participants were recruited and assigned to participant groups based on their survey scores for high risk (HR) for psychosis/prodromal. Participants were categorized as Group 1 (controls) (*n*_1_ = 72) if they scored between 0 and 5 on the questionnaire. Group 2 (HR) (*n*_2_ = 49) scored a 6 or higher on the questionnaire. Participation was voluntary and anonymous and participants were treated in accordance with the “Ethical Principles of Psychologists and Code of Conduct” [27]. Participants received extra credit for participation.

#### 2.1.1.  16-Item Prodromal Questionnaire

The Prodromal Questionnaire (PQ) [28] has been used as screening stage 1 of 2 for psychosis. Ising et al. [28] determined the reliability of the 16-item questionnaire using ROC analysis: 87% sensitivity, 87% specificity, and 44% positive predictive values. Internal consistency reached an alpha level of .77 and achieved congruent validity with the Comprehensive Assessment of At-Risk Mental State (CAARMS, [25]) diagnosis (*r =*.572, *p* < .01, [3]). The 16-item PQ has highly comparable reliability and validity scores compared to the original 92-item PQ and therefore is considered to have met the criteria for use as a screening instrument [28].

The 16-item PQ responses will be coded as 0's for “*false*” and 1's for “*true.*” If participants answer “*true*,” the questionnaire asks for level of severity. The highest possible score on the 16-item PQ was 16 points and the lowest possible score was 0 points. The generally accepted cutoff score is 6 [28]. Therefore, all participants who score a 6 or higher were placed in the high-risk group. The questionnaire scores had a mean of 5.32 (SD = 4.34) for all participants.

### 2.2. Procedure

#### 2.2.1. MMN Paradigm

Brainwaves were recorded using an EEG (NeuroScan 4.0 system). Electrode placements followed the 10-20 system and included the following locations:* Fz, Cz, Pz, *left and right mastoids, lateral and superior of the right eye, a ground electrode from the forehead, and a reference electrode from the tip of the nose. Participants were presented with 2880 samples (120 cycles of 24 samples) of randomized tones (standard = 500 ms; deviant = 250 ms) through headphones for approximately 30 minutes. Participants were instructed to sit quietly in a dark room and view a silent film while tones were presented via headphones. The deviant tones were programmed to occur 8% of the time and the standard tone was presented 92% of the time.

#### 2.2.2. Data Analysis

The recorded signals were separated into epochs (400 ms) with a 100 ms prestimulus interval relative to time pulses. For baseline correction, the average voltage of the 100 ms prestimulus interval was subtracted from each signal trial (tone-onset: time = 0). Artifact rejection was set to ±250 hz and all trials where a channel's voltage exceeds ±75 *μ*V were removed. For each participant, standard and deviant evoked responses were averaged separately; then the standard averages were subtracted from the deviant averages to determine the MMN amplitude. Each participant had a minimum of 100 deviant trials used to create the average deviant waveform. The MMN amplitude was the peak negative amplitude occurring between 140 and 210 ms after the onset of the deviant tone. Based on the definition, MMN must be the most negative point in the valley; therefore, any participants who did not have a negative MMN amplitude at any of the electrode placements during the window of interest were removed (*N* = 121; 67 of original 188 removed). Otherwise, all other participants were included if the recorded data fit the MMN criteria described above.

## 3. Results

**Figure 1:**
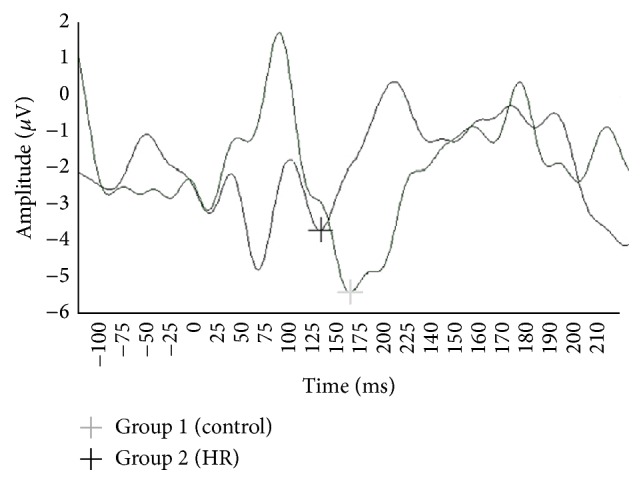
Group amplitude averages for* Cz* electrode location.

A one-way analysis of variance (ANOVA) was used to evaluate mean difference between Group 1 (control) and Group 2 (HR) at each electrode location (*Fz, Cz, *and* Pz*). For* Cz*, Group 1 (control) had a mean amplitude of −5.16 (SD = 2.45) and Group 2 (HR) had a mean of −3.92 (SD = 2.19) (see Figure 1). At* Fz, *Group 1 controls had a mean amplitude of −4.21 (SD = 2.01) and Group 2 (HR) had a mean of −3.35 (SD = 1.88). For* Pz*, Group 1 (control) had a mean amplitude of −3.84 (SD = 1.77) and Group 2 (HR) had a mean of −3.10 (SD = 1.47) (see Table 1). For all electrode locations, healthy controls demonstrated significantly more negative amplitudes than those who qualified as high risk according to the questionnaire (*Cz: F*(1,119) = 8.09, *p* = .005;* Fz*: *F*(1,119) = 5.74, *p* = .018;* Pz*: *F*(1,119) = 5.88, *p* = .017) (see Table 2).

## 4. Discussion

The purpose of the present study was to evaluate the relationship between MMN amplitudes and the 16-item Prodromal Questionnaire's established cutoff scores. The hypothesis was that participants considered at high risk for psychosis would demonstrate more negative amplitudes on average than controls. Results supported this hypothesis, as a statistically significant mean difference in amplitudes at each electrode location was observed.

Current literature focuses on finding correlates of high risk for psychosis as a way to gain insight on the nature of prodromal stages and furthermore identify early stages to provide opportunities for early and more accurate intervention [14]. The most common way to assess degree of psychosis has traditionally been through use of survey research. Although there are multiple types of surveys that analyze symptomology and are used to predict the extent to which an individual will likely convert from healthy to at-risk or from at-risk to diagnosed psychosis, Salisbury et al. [10] findings state that a combination of methods allows for greater accuracy when trying to classify participants. Therefore, the present study sought to combine survey analysis with an electrophysiological component in order to support the use of MMN as an informative tool for distinguishing groups within a homogenous population. Findings of the present study align with the results of prior research [5, 11, 15, 19, 21–23], such that a lower dMMN amplitude was observed in those who met the criteria for HR based on the 16-item PQ.

Although an effect was found, there were limitations to the present study. There was limited diversity across the participant pool and samples were drawn from undergraduate psychology course. All participants were grouped based on self-report. Thus, indication of prior diagnoses of self or immediate family members was up to the discretion of the participants and may result in either intentional or accidental deceit. Participant may not be aware of all family diagnoses or wish not to report family or self-diagnoses due to confidentiality. Alternatively, participants may indicate a diagnosis based on speculation as opposed to a true, clinical diagnosis. It should be noted that this means of assessing diagnoses may have limitations in terms of identifying group membership. The psychometric properties of the questionnaire supported use to identify those who may be at risk for psychosis but do not offer enough empirical basis for a true diagnosis of a current psychotic disorder. However, since an effect was found, findings further supported dMMN as a viable option for detecting subtle differences in a population that would be perceived as healthy and future research will aim to follow participants to identify those who convert into psychosis or schizophrenia to strengthen the predictive qualities of dMMN.

Early interventions to defer the development of psychosis are lacking; thus Nagai et al. [16] argued for the MMN paradigm as a translatable clinical biomarker. Present results supported Nagai et al. [16] and the use of dMMN as an informative, predictive measure to identify individuals who are currently prodromal or may develop psychosis. This practical application may also be informative about the underlying pathology of prodromal stages of psychosis.

There is a growing literature indicating that there may be a variety of other characteristics that accompany high risk for psychosis as measured by a psychological inventory. The current study suggests that neurophysiological correlates may also be a useful tool to accompany psychological screenings for psychosis. While the current study did not longitudinally follow the students to be able to determine which, if any, students experienced a psychotic episode, it did suggest that there may be neural functioning that translates to high endorsement of psychotic symptoms and the brain's ability to detect deviant stimuli in the environment. In addition, the current study sought to use a quick and efficient screen for psychosis in a college population to try to determine if this type of assessment is able to detect those at risk that might otherwise fall through the cracks in a university setting. The results suggest that these students do, in fact, differ from their same age peers who do not endorse symptoms associated with psychosis and that the screening may be a viable way to find students at risk for psychosis. Sommer and colleagues [1] argue that identifying risk for psychosis early in the trajectory of the illness is the key for successful interventions for not only schizophrenia but also other psychiatric disorders that include psychotic symptoms during adolescence [29]. Our results suggest that the use of MMN as a neurophysiological biomarker for psychosis in otherwise healthy young adults is promising and may lead to earlier and more effective interventions.

## Figures and Tables

**Table 1:** Descriptive statistics for groups by electrode location.

Electrode	Group	*N*	Mean	SD	CI lower	CI higher
*Cz*	1	72	−5.16	2.46	−5.74	−4.59
*Cz*	2	49	−3.92	2.19	−4.55	−3.29
*Fz*	1	72	−4.21	2.01	−4.69	−3.74
*Fz*	2	49	−3.35	1.88	−3.89	−2.81
*Pz*	1	72	−3.84	1.77	−4.26	−3.43
*Pz*	2	49	−3.10	1.47	−2.53	−2.67

*Note. *Confidence interval (CI) at the 95% level. Group 1: control; Group 2: high-risk.

**Table 2:** ANOVA table for elecrtode locations.

Electrode	Source	SS	*df*	MS	*F*
*Cz*	Between groups	44.87	1	44.87	8.09^*∗∗*^
	Error	660.01	119	5.55	

	Total	704.88	120		

*Fz*	Between groups	21.96	1	21.96	5.74^*∗*^
	Error	454.92	119	3.82	

	Total	476.88	120		

*Pz*	Between groups	16.19	1	16.19	5.88^*∗*^
	Error	327.58	119	2.75	

	Total	343.77	120		

*Note. ∗* denotes significance at the .05 level; *∗∗* denotes significance at the .01 level.
